# circ_0072464 Shuttled by Bone Mesenchymal Stem Cell-Secreted Extracellular Vesicles Inhibits Nucleus Pulposus Cell Ferroptosis to Relieve Intervertebral Disc Degeneration

**DOI:** 10.1155/2022/2948090

**Published:** 2022-06-29

**Authors:** Xiaojun Yu, Haoran Xu, Qikun Liu, Yingguang Wang, Shanxi Wang, Rui Lu, Yongqiao Jiang, Hao Kang, Weihua Hu

**Affiliations:** Department of Orthopedics, Tongji Hospital, Tongji Medical College, Huazhong University of Science and Technology, Wuhan 430030, China

## Abstract

Ferroptosis, as an iron-dependent form of necrotic cell death, has been reported to affect activities of nucleus pulposus cells (NPCs). However, its role in the pathogenesis of intervertebral disc degeneration (IDD) is largely unknown. Notably, our bioinformatics analysis predicted that circ_0072464 was downregulated in nucleus pulposus of IDD mice. Therefore, this study is aimed at clarifying the mechanisms of extracellular vesicle- (EV-) encapsulated circ_0072464 derived from bone marrow mesenchymal stem cells (BMSCs) in NPC ferroptosis in IDD. EVs were extracted from mouse BMSCs (BMSC-EVs) and then cocultured with IL-1*β*-induced NPCs, followed by detection of matrix synthesis, proliferation, and ferroptosis of NPCs based on gain- or loss-of-function experiments. It was found that the uptake of BMSC-EVs by NPCs alleviated IDD. circ_0072464 and NRF2 were downregulated, and miR-431 was upregulated in IDD. Mechanistically, circ_0072464 competitively bound to miR-431, which targeted and inhibited NRF2 expression. BMSC-derived EVs carrying circ_0072464 inhibited NPC ferroptosis to promote matrix synthesis and proliferation of NPCs by inhibiting miR-431 and upregulating NRF2. Besides, *in vivo* experiments also confirmed that BMSC-EVs alleviated intervertebral disc lesions in mice with IDD through the circ_0072464/miR-431/NRF2 axis. Collectively, BMSC-EV-loaded circ_0072464 inhibited NPC ferroptosis to relieve IDD via upregulation of miR-431-mediated NRF2, therefore providing a potential therapeutic target against IDD.

## 1. Introduction

Intervertebral disc degeneration (IDD), as a multifactor-mediated and age-related disease, is a universal chronic process that can lead to serious spinal symptoms, including low back pain [1]. The cellular and biochemical changes in the microenvironment of intervertebral disc (IVD) occur in the development of IDD, resulting in progressive functional and structural damage [2]. During IDD, mechanical loading may cause extracellular matrix damage, leading to abnormity in microenvironment in nucleus pulposus cells (NPCs) [3]. It has been reported that ferroptosis of NPCs is implicated during the process of IDD [4]. Ferroptosis is a newly studied mechanism of cell death with the essence presented as the disorder of cell lipid metabolism catalyzed by iron ions [5].

New data are accumulating on the beneficial effects of mesenchymal stem cell- (MSC-) derived extracellular vesicles (EVs) in various human diseases, including IDD [6]. EVs (exosomes and microvesicles), as membranous vesicles that are secreted by most cell types, are increasingly considered as important mediators of intercellular communication and biomarkers of diseases [7]. The pathophysiological role of EVs in IDD has been increasingly recognized [8]. Moreover, exosome-transported circular RNAs (circRNAs) have been demonstrated to exert effects on IDD [9]. circRNAs are a large class of endogenous noncoding RNAs, whose 5′ and 3′ ends are connected together to form a closed-loop structure, and play a vital role in various pivotal biological processes, including cell proliferation, apoptosis, and metabolism [10].

In addition, circRNAs, as a competitive endogenous RNA (ceRNA), competitively bind to microRNAs (miRNAs) to regulate the expression of miRNA target messenger RNA (mRNA), which serves as a potential biomarker for IDD treatment [11]. miRNAs, as small noncoding RNAs, affect some biological processes, such as apoptosis, by regulating the target gene expression [12]. Existing evidence has revealed that dysregulated miRNA expression is involved in the pathogenesis of IDD [12, 13]. Our bioinformatics analysis prior to investigations revealed putative binding relation between circ_0072464 and miR-431. It is interesting to note that miR-431, negatively regulated by circSEMA4B, modulates the progression of IDD [14]. However, the mechanism by which BMSC-derived EV communication affects NPC ferroptosis in IDD involving the interplay between circ_0072464 and miR-431 is still poorly understood.

Hence, we hypothesized that the transfer of circ_0072464 via BMSC-derived EVs (BMSC-EVs) might affect NPC ferroptosis in IDD.

## 2. Methods

### 2.1. Ethics Statement

The study protocols were approved by the Ethics Committee of Tongji Hospital, Tongji Medical College, Huazhong University of Science and Technology. All animal procedures were in line with the *Guide for the Care and Use of Laboratory* animals published by National Institutes of Health.

### 2.2. Isolation and Identification of BMSCs

Bone marrow tissues were collected from 3 normal C57BL/6 mice (age: 8 weeks and weight: 18-20 g), and BMSCs were isolated from mice as previously described [15–17]. BMSCs were cultured in DMEM/F12 (HyClone Company, Logan, UT) supplemented with 10% fetal bovine serum (FBS, Gibco, Grand Island, NY) and 0.2% penicillin and streptomycin (HyClone). The medium was renewed once every three days, and the BMSCs after the 3^rd^-7^th^ medium renewal were used for further experiments.

In addition, BMSCs were cultured in OriCell™ MSC osteogenic, adipogenic, or chondrogenic differentiation medium (Cyagen Biosciences Inc., Guangzhou, China), respectively. Next, Alizarin red staining was used to observe calcium deposition on the 14^th^ day of induction, oil red O staining to observe lipid droplets on the 21^st^ day of induction, and Alcian blue staining to observe extracellular matrix on the 28^th^ day of induction.

### 2.3. Isolation and Characterization of BMSC-EVs

BMSCs were ultracentrifuged at 100000 × g at 4°C in the medium containing serum overnight to remove EVs. Next, BMSCs were cultured in conditioned medium (DMEM/F12+10% EV-free serum) for 48 h. The culture supernatant of BMSCs in logarithmic growth phase was collected to extract EVs. BMSCs were centrifuged (500 × g) for 10 min at 4°C to collect supernatant, which was repeated one time. BMSCs were then centrifuged (2000 × g, 30 min) to remove large vesicle, centrifuged (10000 × g, 20 min, 4°C) to remove cellular debris, filtered using a 0.22 *μ*m filter, and centrifuged (110000 × g, 70 min) again. At last, precipitate was resuspended by PBS, centrifuged, resuspended again with 100 *μ*L sterile PBS, and stored at -20°C.

The diameter distribution of BMSC-EVs was assessed by nanoparticle tracking analysis (NTA) based on Nanosizer™ technology (Malvern Instruments, Malvern, UK). The morphology of the acquired EVs was observed under the transmission electron microscopy (TEM; Tecnai 12, Philips, Netherlands). Western blot analysis was used to examine the specific EV surface markers, including anti-CD63 (1: 2000, #ab216130, Abcam), anti-tumor susceptibility gene 101 (TSG101, 1: 10000, #ab125011, Abcam), anti-CD81 (1: 10000, #ab109201, Abcam), and anti-calnexin (1: 100000, #ab92573, Abcam). Protein content of BMSC-EVs was determined by a bicinchoninic acid (BCA) assay (Thermo Fisher Scientific, Waltham, MA) with the absorbance read at 562 nm using a microplate reader.

### 2.4. Surgically Induced IDD Mouse Model

Fifty C57BL/6 male mice (aged 8 weeks) provided by Beijing Vital River Laboratory Animal Technology Co., Ltd., (Beijing, China) were raised under specific pathogen-free conditions (22°C, 50%-55% humidity, and 12 h light/dark cycle). The specific procedures were performed as previously reported method [18]. The Co6-Co7 discs were collected at week 6 and 12 after surgery from the mice for subsequent experiments.

Mice were divided into control group (*n* = 10) and IDD group (*n* = 40). Next, IDD mice (*n* = 40) were injected with PBS, BMSC-EVs (200 *μ*g), ferroptosis inducer Erastin (50 mg/kg, EMD Millipore Corp., Billerica, MA), or Erastin+EVs (200 *μ*g BMSC-EVs and 50 mg/kg Erastin) via tail vein (*n* = 10). The mice were injected every 2 days until they were euthanized.

### 2.5. Safranin O Staining

The discs from mice were fixed in 10% neutral-buffered formalin for 1 week, decalcified in EDTA for 2 weeks, embedded by paraffin, and sliced into sections (5 *μ*m). The sections were stained with eosin and safranin O-fast green. Then, the Olympus BX51 microscope (Olympus Center Valley, PA) was employed to analyze histological images. The cellularity and morphology of the annulus fibrosus (AF), nucleus pulposus (NP), and the border between the two structures were examined. Three pathologists scored independently according to the scoring criteria of histological sections [18].

### 2.6. X-Ray Examination

At weeks 6 and 12 after puncture, radiographs were conducted. The disc height index (DHI) was applied to evaluate IVD height. The control discs and the corresponding punctured discs were measured. The heights of disc and the adjacent vertebral body were measured on the midline, and 25% of the disc width on both sides was measured from the midline. DHI was presented as the mean value of the three measurements from the midline to the 50% of disc width boundary at the center divided by the average of the two adjacent vertebral body heights. The change of DHI in puncture disc was displayed as %DHI = postpunctured DHI/prepunctured DHI × 100.

### 2.7. Immunofluorescence Staining

Frozen sections of discs from IDD mice were fixed with 4% paraformaldehyde for 5 min and washed with TBST, followed by incubation with 10 mg/mL hyaluronidase (Sigma-Aldrich Chemical Company, St Louis, MO) at 37°C for 30 min. The sections were then sealed with 10% goat normal serum (Nichirei Biosciences, Tokyo, Japan) and cultured with primary antibodies (anti-matrix metallopeptidase 13 (MMP13, 1: 2000, #ab219620, Abcam) and anti-Col II (1: 2000, #ab34712, Abcam)) for 2 h at room temperature. The sections embedded with paraffin received deparaffinization and incubation in 1 mM EDTA (pH 8.0) at 80°C for 15 min for antigen retrieval. The sections were cultured with hyaluronidase (10 mg/mL) at 37°C for 30 min. Furthermore, secondary antibodies were utilized to detect immune complexes. At last, nucleus was stained by DAPI (Invitrogen, Carlsbad, CA).

### 2.8. RNA Extraction and Sequencing

NP tissues were collected from three IDD mice and three normal mice. There are three samples in each group. Total RNAs were extracted with Trizol (Invitrogen). The concentration of RNA was detected using Nanodrop ND-1000 spectrophotometer (Thermo Fisher) through optical density (OD)260/280. Qubit RNA Assay Kit was used to detect RNA concentration. Total RNA samples with RNA integrity number (RIN) ≥ 7.0 and a 28S : 18S ratio ≥ 1.5 were selected for the following experiments.

CapitalBio Technology (Beijing, China) was adopted to generate and sequence the sequencing library with 5 *μ*g RNA per sample. The detailed methods were in line with previously published study [19]. Finally, the paired-end sequencing was performed on an Illumina NextSeqCN500 sequencer.

### 2.9. Quality Control and Comparison

The quality of paired-end reads of the original sequencing data was checked using FastQC software v0.11.8. The Cutadapt software 1.18 was utilized to pretreat original data to remove Illumina sequencing connector and poly (A) tail sequence. Perl script was used to remove reads with N more than 5%. FASTX Toolkit software 0.0.13 was employed to extract reads with 70% base mass above 20. Double-ended sequence was repaired by the BBMap software. Finally, the filtered high-quality read fragments were compared with the human reference genome by the bwa software (0.7.12).

### 2.10. Bioinformatics Data

The CIRI software [20] was adopted to identify reverse splicing events, and sequencing data that cannot be directly compared with the reference genome were used for subsequent circRNA analysis. The screened circRNA was annotated using circBase website. The differentially expressed circRNAs were analyzed by limma (v3.32.10) package of R software [21] with |logFC| > 1 and *p* value < 0.05.

The Circinteractome database was utilized to predict the key circRNAs that regulate miRNA. The downstream target genes of candidate miRNA were predicted by RNA22 database and miRDB database. Next, IDD-related mRNA data GSE56081 was retrieved from GEO database. This microarray dataset included 5 IDD samples and 5 control samples. The differentially expressed mRNAs were screened with |logFC| > 0.2 and *p* value < 0.01. The “limma” package of R language was utilized for differential analysis. The predicted results of RNA22 and miRDB database were intersected with downregulated mRNA in the IDD sample of GSE56081 to screen the target genes regulated by the downstream of candidate miRNA.

### 2.11. Isolation of NPCs and IDD Cell Model Establishment

NP tissues were collected from 3 normal C57BL/6 mice (age: 8 weeks and weight: 18-20 g), washed with PBS, and sliced into sections, which were treated with 0.25% streptomycin protease (Sigma-Aldrich) for 30 min. After digestion with 0.2% type II collagenase (Roche, Indianapolis, IN) for 4 h, cells were cultured in DMEM/F12 containing 10% FBS and 100 U/mL penicillin-streptomycin in a humidified atmosphere of 5% CO_2_. NPCs at passage 3-5 *in vitro* were identified by immunofluorescence assay using rabbit antibodies against Brachyury/T (1 : 1000, #ab209665, Abcam), Cytokeratin 19 (1 : 500, #ab52625, Abcam), and secreted frizzled related protein 2 (SFRP2) (1 : 500, PA5-106726, Invitrogen), among which Brachyury/T and Cytokeratin 19 are proteins positive for NPCs while SFRP2 was used as a negative control (NC) (Figure S1). IL-1*β* (10 ng/mL) was added to NPCs with purity > 95% to establish IDD cell model *in vitro*.

### 2.12. Lentiviral Transduction and Grouping

Lentivirus vectors (short hairpin RNA against circ_0072464 (sh-circ_0072464), overexpressed circ_0072464 (oe-circ_0072464), oe-NRF2, miR-431 mimic, and miR-431 inhibitor) and control lentivirus vector (sh-NC, oe-NC, mimic NC, and inhibitor NC) (Shanghai Genechem Co., Ltd., Shanghai, China) were purified and cotransfected with packaging vector into 293FT cells (ATCC, Manassas, VA). After 48 h, cell supernatant was collected. Lentivirus particles in the supernatant were concentrated at a ratio of 1 : 100 by ultracentrifugation and suspended in PBS for recovery. Virus titer was determined by enzyme-linked immunosorbent assay Kit (Takara, Kusatsu, Japan). Then, virus (1 × 10^8^ TU/mL) was incubated with BMSCs or NPCs to obtain stably transduced cell lines.

1. Stably transduced BMSCs were obtained to extract EVs (EVs-sh-NC and EVs-sh-circ_0072464)
2. NPCs were treated with PBS or cocultured with EVs, EVs-sh-NC, or EVs-sh-circ_0072464
3. NPCs were stably transduced with oe-NC, sh-NC, oe-circ_0072464, sh-circ_0072464, mimic NC, inhibitor NC, miR-431 mimic, miR-431 inhibitor, or sh-NRF2 or treated with Erastin (10 *μ*M).

### 2.13. Uptake of EVs by NPCs

EVs were labeled with green fluorescent dye (PKH67; Sigma-Aldrich) and then cocultured with Hoechst-labeled NPCs at 37°C for 2 h and fixed in 4% paraformaldehyde for 15 min. Observation of nitracellular green fluorescence was performed with the help of a fluorescence microscope (CX23, Olympus, Tokyo, Japan). Then, EV-stimulated receptor cells were collected, and circ_0072464 expression was determined by reverse transcription quantitative polymerase chain reaction (RT-qPCR).

### 2.14. Cell Counting Kit-8 (CCK8) and EdU Assays

Cell viability of NPCs was detected according to the instructions of CCK8 (Dojindo Molecular Technologies, Inc., Kumamoto, Japan). The OD values were measured at the wavelength of 450 nm.

EdU assay was carried out based on previous study [22]. The EdU positive rate was calculated: EdU positive rate = EdU − positive cells (red cells)/total Hoechst 33258 − positive cells (blue cells).

### 2.15. Immunofluorescence

The NPCs were subjected to fixation in 4% paraformaldehyde, permeabilization with PBS containing 0.25% Triton X-100 for 10 min, and blocking with 4% bovine serum albumin (BSA) containing 0.25% Triton X-100 for 30 min. Then, cells were incubated with primary antibodies rabbit anti-Col II (1 : 2000, #ab34712, Abcam), rabbit anti-MMP13 (1 : 2000, #ab219620, Abcam), rabbit anti-Brachyury/T (1 : 1000, #ab209665, Abcam), rabbit anti-Cytokeratin 19 (1 : 500, #ab52625, Abcam), and rabbit anti-SFRP2 (1 : 500, PA5-106726, Invitrogen) overnight at 4°C. After washing, the cells were subjected to incubation with goat anti-rabbit IgG (H&L) conjugated with Alexa Fluor 488 (1 : 500, #ab150077, Abcam). Next, the nuclei were counterstained with DAPI (Invitrogen) for 5 min. CarlZeiss LSM710 confocal microscope (CarlZeiss, Oberkochen, Germany) was used to visualize the fluorescence with images analyzed by the Image-Pro Plus 6.0 software (Media Cybernetics, Silver Spring, MD).

### 2.16. RNA Fluorescent In Situ Hybridization (FISH)

After transduction with circ_0072464 and miR-431 expressing vectors, NPCs or NP tissue sections were subjected to FISH assay [23]. The IX-53 microscope (Olympus, Tokyo, Japan) was employed to obtain images.

#### 2.16.1. Immunohistochemistry

Antigen was retrieved from paraffin NP tissue sections (4 *μ*m) by boiling in EDTA buffer solution (0.05 mol/L Tris and 0.001 mol/L EDTA; pH 8.5) in a microwave oven for 20 min. The tissue sections were blocked with 5% BSA for 20 min and incubated with primary antibodies against NRF2 (1 : 200, PA5-88084, Thermo Fisher Scientific), GPX4 (1 : 200, ab125066, Abcam), and ACSL4 (1 : 200, ab155282, Abcam) overnight at 4°C, followed by incubation with biotinylated goat anti-rabbit IgG secondary antibody (1 : 2500, ab205718, Abcam) for 20 min. Thereafter, the sections were exposed to DAB substrate and counterstained with haematoxylin. Finally, the sections were photographed under a microscope (Leica-DM2500, Leica, Wetzlar, Germany). The ImagePro Plus 7.1 software (Media Cybernetics, Silver Spring, MD) was applied for quantitative analysis.

### 2.17. BODIPY Staining

Lipid reactive oxygen species (ROS) levels were measured by C11-BODIPY 581/591 (Thermo Fisher, D3861). Specifically, the cells were incubated with 10  *μ*M C11-BODIPY 581/591 at 37°C for 30 min. After washing with PBS three times, cells were detached with trypsin and resuspended in fresh PBS. A flow cytometer (BD Biosciences) was utilized to analyze fluorescence emission peaks. The peak from 590 nm to 510 nm is inversely proportional to the production of lipid ROS.

### 2.18. Measurement of Labile Iron Contents

Based on the instructions, an iron assay kit (ab83366, Abcam) was applied for iron concentration detection. Next, cells were treated with various compounds, collected, and homogenized by 5 times the volume of iron assay solution on ice, followed by centrifugation (13,000 × g, 10 min) at 4°C to obtain the supernatant. Cells were incubated with iron reducer (5 *μ*L) for 30 min at 37°C and then with iron probe (100 *μ*L) for 60 min at 37°C in the dark. Absorbance value was measured using a microplate reader (Thermo Fisher) at the wavelength of 593 nm.

### 2.19. TEM

Cells were fixed in 2.5% glutaraldehyde (Sigma-Aldrich) for 1 h and in 2% osmium tetroxide for 3 h. Then, cells were washed, stained with 0.5% uranyl acetate for 12 h, dehydrated, polymerized, and sliced into sections (70-90 nm) with an ultramicrotome (EM UC7, Leica), followed by observation under a TEM (FEI, USA).

### 2.20. RT-qPCR

Total RNAs were extracted using the miRNeasy Mini Kit (Qiagen, Venlo, The Netherlands). RNA was reversely transcribed using PrimeScipt RT Kit (Takara). miR-431 was reversely transcribed using specific tailing reaction kit (B532451, Sangon, Shanghai, China). RT-qPCR was carried out on the 7500 Sequence Detection System (ABI, Foster City, CA) using SYBR Premix Ex Taq (Takara). The 2^-*ΔΔ*Ct^ method was used to quantify relative expression levels of target genes. For circ_0072464, total RNAs were incubated with or without 3 U/*μ*g of RNase R (Epicentre, San Diego, CA) at 37°C for 20 min, and the resultant RNA was subsequently purified using the RNeasy MinElute Cleanup Kit (Qiagen). The circRNA was amplified using the specific divergent primers for the back-splice junction of circ_0072464. The agarose gel electrophoresis and sequencing were conducted to detect the amplified products. U6 and *β*-actin served as internal reference for miRNA and mRNA, respectively. All primers are shown in Table S1.

### 2.21. Western Blot Analysis

Total protein was extracted using radioimmunoprecipitation assay (RIPA) lysis buffer (R0010, Solarbio Science & Technology Co., Ltd., Beijing, China) containing PMSF, and protein contents were measured using the Micro BCA Protein Assay Kit (Thermo Fisher). The lysates were centrifuged and separated by sodium dodecyl sulfate-polyacrylamide gel electrophoresis gels, electrotransferred onto a polyvinylidene fluoride membrane. The membranes were blocked and incubated with the specific primary antibody overnight at 4°C. After washing with TBST, the membrane was subjected to incubation with horseradish peroxidase- (HRP-) labeled anti-rabbit IgG (1 : 1000, #7074, Cell Signaling Technology, Beverly, MA) or HRP-labeled anti-mouse IgG (1 : 1000, #7076, Cell Signaling Technology) for 2 h. The enhanced chemiluminescence reagents (Amersham, Piscataway, NJ) and ChemiDoc XRS system (Bio-Rad, Hercules, CA) were adopted to visualize protein expression. The primary antibodies were as follows: anti-NRF2 (1 : 1000, #ab92946, Abcam), anti-Col II (1 : 400, #ab34712, Abcam), anti-Aggrecan (1 : 1000, #ab36861, Abcam), anti-ADAMTS-5 (1 : 250, #ab41037, Abcam), anti-MMP13 (1 : 500, #ab39012, Abcam), anti-ACSL4 (1 : 10000, # ab155282, Abcam), anti-GPX4 (1 : 1000, #ab125066, Abcam), anti-FTL (1 : 10000, #ab109373, Abcam), and anti-*β*-actin (1 : 2000, #4967, Cell Signaling Technology).

### 2.22. Dual-Luciferase Reporter Gene Assay

The binding sites between circ_0072464 and miR-431, miR-431, and NRF2 were obtained through bioinformatics analysis. Mutations were generated using the QuikChange Site-Directed Mutagenesis Kit (Stratagene, La Jolla, CA). The wild-type NRF2-3′UTR (NRF2-3′UTR-WT), mutant NRF2-3′UTR (NRF2-3′UTR-MUT), circ_0072464-WT, or circ_0072464-MUT were inserted into the Pmel and Xhol or Xba1 restriction sites of pmirGLO luciferase vector (Promega, Madison, WI). NPCs were seeded into 96-well plates at 8 × 10^3^ cells/well and cotransfected with 0.2 *μ*g reporter plasmid and 40 nM miR-431 mimic or mimic NC using Opti-MEM (Invitrogen) and Lipofectamine 3000. After 48 h, the Dual-Luciferase Reporter Assay System (Promega) was utilized to measure luciferase activity. The OD value of solution was measured using the SpectraMax i3x multiplate reader (Molecular Devices, Sunnyvale, CA). The luciferase activity was calculated as the ratio of firefly and Renilla luciferase activity.

### 2.23. RNA Immunoprecipitation (RIP) Assay

RIP kit (17-701, Millipore) was utilized to detect the binding of NRF2/circ_0072464 and AGO2 protein. Upon cell confluence reached 80% -90%, the medium was removed, and the cells were washed with cold PBS (1 mL) and subjected to RIPA lysis (P0013B, Beyotime Institute of Biotechnology, Shanghai, China) on ice bath for 5 min and centrifugation at 32876.4 × g at 4°C for 10 min. A part of cell extraction liquid served as an input, and another part was cultured with antibody for coprecipitation. In brief, the magnetic bead (50 *μ*L) was suspended with RIP Wash Buffer (100 *μ*L) and incubated with 5 *μ*g antibody rabbit anti-mouse AGO2 (1: 100, ab32381, Abcam) or goat anti-mouse IgG (1 : 100, ab205719, Abcam) at room temperature for 30 min. The magnetic bead-antibody compound was suspended with RIP Wash Buffer (900 *μ*L) and incubated with cell extraction liquid (100 *μ*L) at 4°C. The sample was placed on the magnetic base to collect the complex of magnetic bead-protein. The sample and input were detached by protease K to extract RNA for RT-qPCR to determine NRF2 and circ_0072464.

### 2.24. RNA Pull-Down Assay

NPCs were transfected with biotin-labeled Bio-NC, Bio-miR-431-WT and Bio-miR-431-MUT RNA or Bio-NRF2-WT and Bio-NRF2-MUT RNA (50 nM) for 48 h. After washing with PBS, NPCs were incubated with lysis buffer containing protease inhibitor (Roche) and ribonuclease inhibitor (Fermentas, St. Leon-Rot, Germany) (20 mM Tris, pH 7.5, 200 mM NaCl, 2.5 mM MgCl_2_, 0.05% IGEPAL CA-630, and 1 mM dithiothreitol) on ice for 10 min. The lysate was cultured with magnetic beads coated by streptavidin (M-280, Invitrogen) at 4°C overnight. The RNase free BSA (Sigma-Aldrich) and yeast tRNA (Sigma-Aldrich) were used to precoat magnetic beads. Expression of circ_0072464 and miR-431 was detected by RT-qPCR.

### 2.25. Statistical Analysis

All data were presented as mean ± standard deviation and analyzed by the SPSS 21.0 statistical software (IBM Corp., Armonk, NY). The normality and homogeneity of variance test were conducted. Comparisons of data between two groups were analyzed by independent sample *t-*test, and those among multiple groups were tested by one-way analysis of variance (ANOVA) or repeated measures ANOVA, followed by Tukey's post hoc test. *p* < 0.05 was regarded as statistically significant.

## 3. Results

### 3.1. BMSC-EVs Alleviated IDD

BMSCs were isolated from bone marrow, and EVs were extracted from BMSCs, both of which were well characterized. Isolated BMSCs under ordinary microscope showed fibrous aggregation growth, and Alizarin red staining, oil red O staining, and Alcian blue staining further confirmed that the isolated BMSCs had the potential to differentiate into osteoblasts, adipoblasts, and chondroblasts (Figure S2A-B). These findings suggested that BMSCs were successfully isolated.

**Figure 1:**
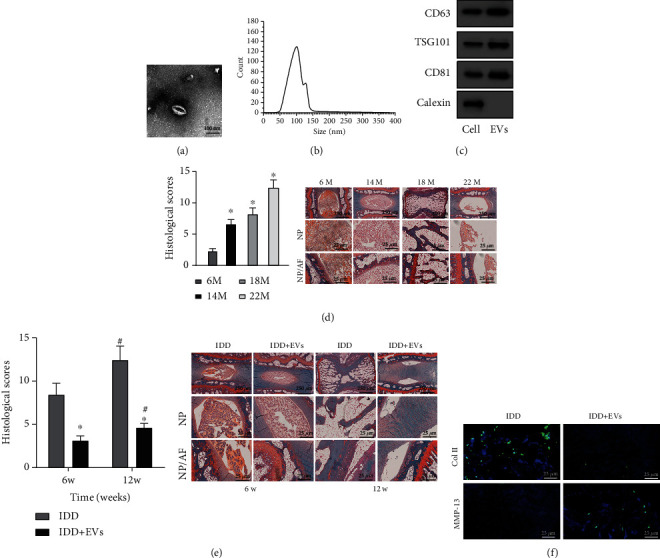
Effects of BMSC-EVs on IDD mice. (a) The morphology of EVs observed under the TEM. (b) The particle size distribution of EVs analyzed by NTA. (c) The protein levels of EV markers CD63, TSG101, and CD81 and negative control calnexin detected by Western blot analysis. (d) The NP and cartilage AF of IVD after modeling detected by safranin O staining (*n* = 10). The first row shows the staining image of IVD as a whole, the second row displays the detailed view of NP tissues, and the third row presents the detailed view of boundary between NP and AF tissues. ^∗^*p* < 0.05*vs.* IDD mice at the 6^th^ month (M). (e) The histopathology of NP and the AF of NP and cartilage detected by safranin O staining (*n* = 10). The first row shows the staining image of IVD as a whole, the second row displays the detailed view of NP tissues, and the third row presents the detailed view of boundary between NP and AF tissues. ^∗^*p* < 0.05*vs.* IDD mice and ^#^*p* < 0.05*vs.* IDD mice at the 6^th^ week (W). (f) The levels of Col II and MMP13 in NP tissues measured by immunofluorescence staining (*n* = 10). Cell experiments were repeated for three times independently.

Next, TEM showed that EVs were irregularly circular (Figure 1(a)). In addition, NTA presented that the average diameter of EVs was 110 nm (Figure 1(b)). Western blot analysis exhibited that the levels of CD63, TSG101, and CD81 in EVs were higher than those in cell lysate, and there was no calnexin expression (Figure 1(c)). It can be concluded that EVs were successfully extracted from BMSCs.

Subsequently, Alizarin red staining displayed that as mice grew older, the IVD of mice showed morphological disorder of NP and AF, which indicated that mice had IDD (Figure 1(d)). After successful establishment of IDD mouse models, safranin O staining displayed that compared with IDD mice at the 6^th^ week, the degree of IDD in mice aggravated significantly at the 12^th^ week, and compared with IDD mice, the degree of IDD in IDD mice injected with EVs decreased significantly at the 6^th^ or 12^th^ week (Figure 1(e)). MMP13 and Col II are critical factors reflecting matrix synthesis in the disc during IDD [24, 25]. Immunofluorescence staining revealed that MMP13 level decreased and Col II level increased in IDD mice injected with EVs at the 12^th^ week (Figure 1(f)).

The obtained data suggested that BMSCs and BMSCs-EVs were successfully isolated, and BMSCs-EVs effectively relieved IVD lesions in IDD mice.

### 3.2. circ_0072464 Shuttled by BMSC-EVs Promoted Matrix Synthesis and Proliferation of NPCs

**Figure 2:**
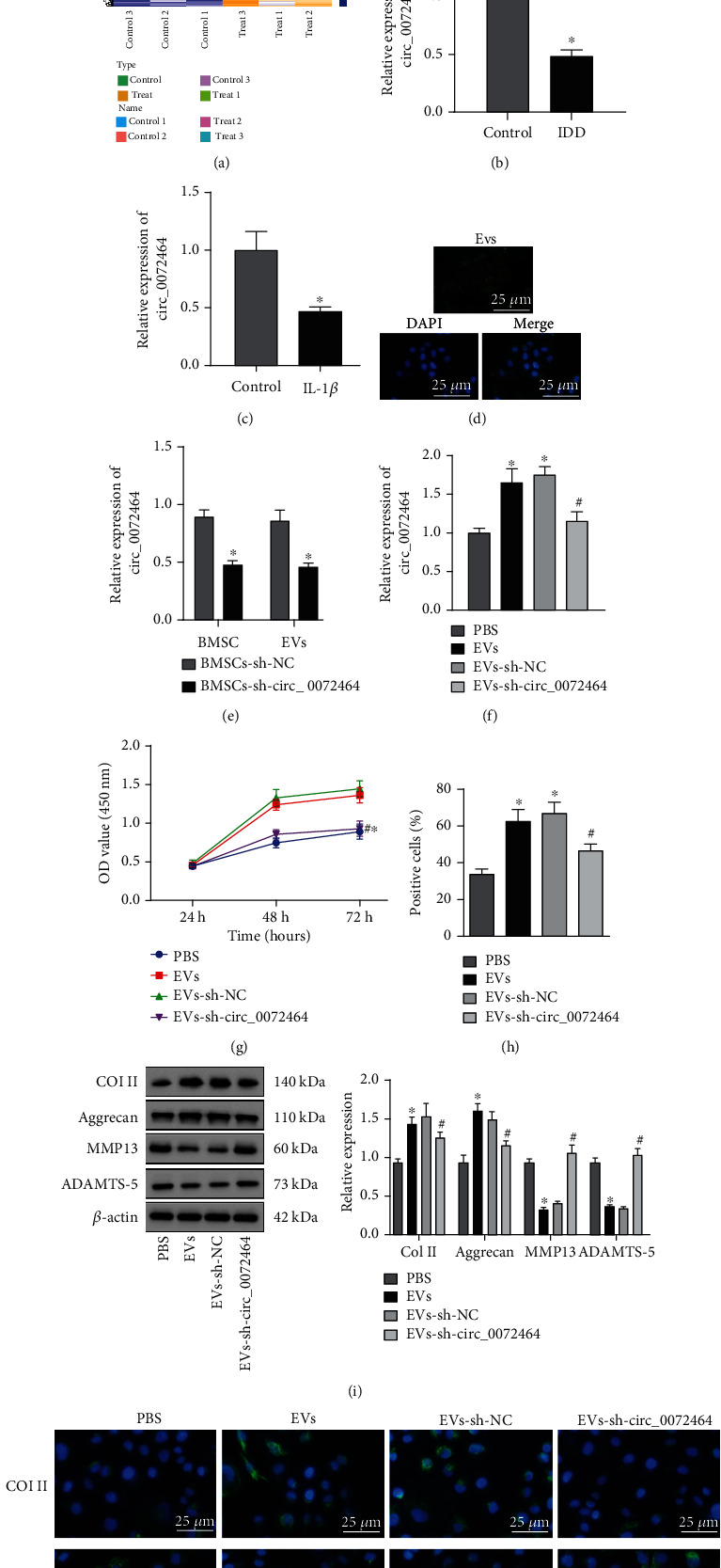
circ_0072464 shuttled by BMSC-EVs affects matrix synthesis and proliferation of NPCs. (a) Heat map of differentially expressed circRNAs screened from RNA-seq data (GSE189551) of NP tissues of IDD mice and normal mice. (b) circ_0072464 expression in NP tissues of IDD mice and normal mice (*n* = 10, ^∗^*p* < 0.05*vs.* control mice). (c) circ_0072464 expression in NPCs determined by RT-qPCR (^∗^*p* < 0.05*vs.* control NPCs). (d) The uptake of EVs by NPCs observed under the confocal microscope; blue: Hoechst staining; green: PKH67-labeled EVs. (e) circ_0072464 expression in BMSC-EVs in response to sh-circ_0072464 determined by RT-qPCR (^∗^*p* < 0.05*vs.* BMSCs transduced with sh-NC). (f) circ_0072464 expression in NPCs after coculture with EVs or EVs-sh-circ_0072464 determined by RT-qPCR. (g) Viability of NPCs after coculture with EVs or EVs-sh-circ_0072464 detected by CCK8 assay. (h) Proliferation of NPCs after coculture with EVs or EVs-sh-circ_0072464 detected by EdU assay. (i) Protein levels of Col II, Aggrecan, MMP13, and ADAMTS-5 in NPCs after coculture with EVs or EVs-sh-circ_0072464 measured by Western blot analysis. (j) Expression of Col II and MMP13 in NPCs after coculture with EVs or EVs-sh-circ_0072464 determined by immunofluorescence staining. In (f–j), ^∗^*p* < 0.05*vs.* PBS-treated NPCs and ^#^*p* < 0.05*vs.* NPCs cocultured with EVs-sh-NC. Cell experiments were repeated for three times independently.

Through sequencing (GSE189551) the NP tissues of IDD mice and control mice, 233 upregulated and 317 downregulated circRNAs were obtained, and circ_0072464 expression decreased in the NP tissues from IDD mice (Figure 2(a)), suggesting that circ_0072464 may play an important role in the development of IDD.

RT-qPCR also exhibited that circ_0072464 was poorly expressed in NP tissues from IDD mice (Figure 2(b)), which was further verified in NPCs that circ_0072464 was also poorly expressed in IL-1*β*-treated NPCs (Figure 2(c)).

Next, the effects of BMSC-EVs carrying circ_0072464 on IDD were explored. Confocal microscope exhibited that BMSC-EVs were internalized by NPCs (Figure 2(d)). Next, BMSCs were transduced with sh-circ_0072464, from which EVs were extracted. RT-qPCR revealed that circ_0072464 expression reduced in BMSCs and EVs after circ_0072464 knockdown (Figure 2(e)). Subsequently, EVs were cocultured with NPCs. RT-qPCR displayed that circ_0072464 expression increased in NPCs cocultured with EVs, and after silencing of circ_0072464, circ_0072464 expression decreased in NPCs (Figure 2(f)). These results confirmed that BMSC-EVs delivered circ_0072464 into NPCs.

Moreover, the roles of BMSC-EVs carrying circ_0072464 in cellular function of NPCs were investigated. CCK8 and EdU assay revealed that cell proliferation of NPCs cocultured with EVs was promoted, yet inhibited after silencing of circ_0072464 (Figures 2(g) and 2(h)). Western blot analysis and immunofluorescence staining showed that levels of Col II and Aggrecan increased, and levels of MMP13 and ADAMTS-5 reduced in NPCs cocultured with EVs, while circ_0072464 knockdown exerted opposite effects (Figures 2(i) and 2(j)).

It can be concluded that BMSC-EVs delivered circ_0072464 into NPCs to promote matrix synthesis and proliferation of NPCs.

### 3.3. circ_0072464 Competitively Bound to miR-431

**Figure 3:**
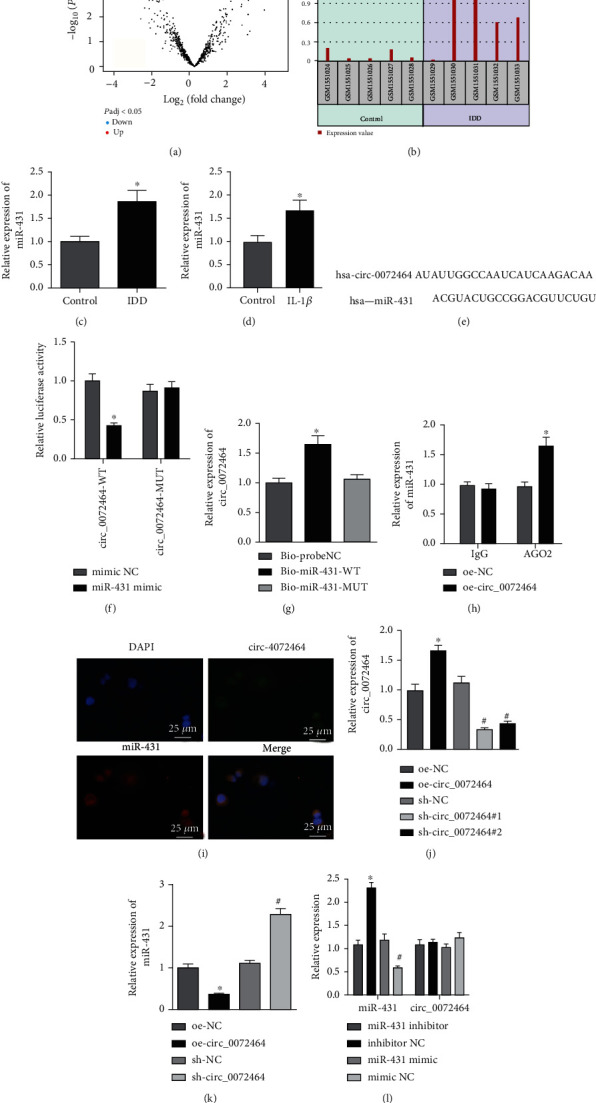
circ_0072464 sponges miR-431. (a) miR-431 expression in IDD sample of GSE63492 dataset. (b) Volcano map of differential analysis for GSE63492 dataset. (c) miR-431 expression in NP tissues of IDD mice determined by RT-qPCR (*n* = 10, ^∗^*p* < 0.05*vs.* control mice). (d) miR-431 expression in IL-1*β*-treated NPCs determined by RT-qPCR (^∗^*p* < 0.05*vs.* control NPCs). (e) The binding site between circ_0072464 and miR-431 predicted by bioinformatics website. (f) The relationship between circ_0072464 and miR-431 verified by dual-luciferase reporter gene assay (^∗^*p* < 0.05*vs.* mimic NC). (g) The interaction between circ_0072464 and miR-431 detected by RNA pull-down assay (^∗^*p* < 0.05*vs.* Bioprobe NC or Bio-miR-431-MUT). (h) The interaction between circ_0072464 and miR-431 detected by RIP assay (^∗^*p* < 0.05*vs.* oe-NC). (i) The colocalization between circ_0072464 and miR-431 in NPCs detected by RNA FISH assay. (j) circ_0072464 expression in NPCs in response to oe-circ_0072464 or sh-circ_0072464 determined by RT-qPCR (^∗^*p* < 0.05*vs.* NPCs transduced with oe-NC; ^#^*p* < 0.05*vs.* NPCs transduced with sh-NC). (k) miR-431 expression in NPCs in response to oe-circ_0072464 or sh-circ_0072464 determined by RT-qPCR (^∗^*p* < 0.05*vs.* NPCs transduced with oe-NC; ^#^*p* < 0.05*vs.* NPCs transduced with sh-NC). (l) Expression of circ_0072464 and miR-431 in NPCs in response to miR-431 inhibitor or mimic determined by RT-qPCR (^∗^*p* < 0.05*vs.* NPCs transfected with mimic NC; ^#^*p* < 0.05*vs.* NPCs transfected with inhibitor NC). Cell experiments were repeated for three times independently.

The downstream mechanism of circ_0072464 was further studied, and the downstream regulatory miRNAs of circ_0072464 were predicted by Circinteractome database, results of which presented that circ_0072464 targeted miR-431. In addition, IDD-related mRNA expression profile GSE63492 was retrieved from GEO database for differential analysis. circ_0072464 and miR-431 are highly conservative in both humans and mice. The NCBI-BLAST comparative analysis indicated that circ_0072464, also known as circARL15, is encoded by ARL15, sequences of which had strong homology in both humans and mice (Figure S3A). Similar results were also detected regarding miR-431 (Figure S3B). The results showed that miR-431 was overexpressed in patients with IDD (Figures 3(a) and 3(b)). RT-qPCR also exhibited that miR-431 expression increased in NP tissues of IDD mice (Figure 3(c)), and in IL-1*β*-treated NPCs (Figure 3(d)).

Next, we moved to illuminate the roles of circ_0072464 in the regulation of miR-431. Bioinformatics website predicted that there was a binding site between circ_0072464 and miR-431 (Figure 3(e)), which was validated by dual-luciferase reporter gene assay (Figure 3(f)). It was found that the luciferase activity of circ_0072464-WT was suppressed by restoring miR-431, while no evident difference was found regarding luciferase activity of circ_0072464-MUT. RNA pull-down and RIP assays confirmed the interaction between circ_0072464 and miR-431 (Figures 3(g) and 3(h)). In addition, RNA FISH assay showed that there was colocalization between circ_0072464 and miR-431 in NPCs (Figure 3(i)).

RT-qPCR exhibited that circ_0072464 expression elevated in NPCs transduced with oe-circ_0072464, while reduced in NPCs transduced with sh-circ_0072464#1 and significantly decreased in NPCs transduced with sh-circ_0072464#2. Thus, sh-circ_0072464#2 (sh-circ_0072464) was selected for further experiments (Figure 3(j)). Moreover, miR-431 expression was decreased in NPCs transduced with oe-circ_0072464, but it was increased in NPCs transduced with sh-circ_0072464 (Figure 3(k)), while mimic or inhibitor of miR-431 exerted no effects on circ_0072464 expression (Figure 3(l)).

The obtained data indicated that circ_0072464 sponged miR-431 to inhibit its expression.

### 3.4. miR-431 Targeted and Inhibited NRF2

**Figure 4:**
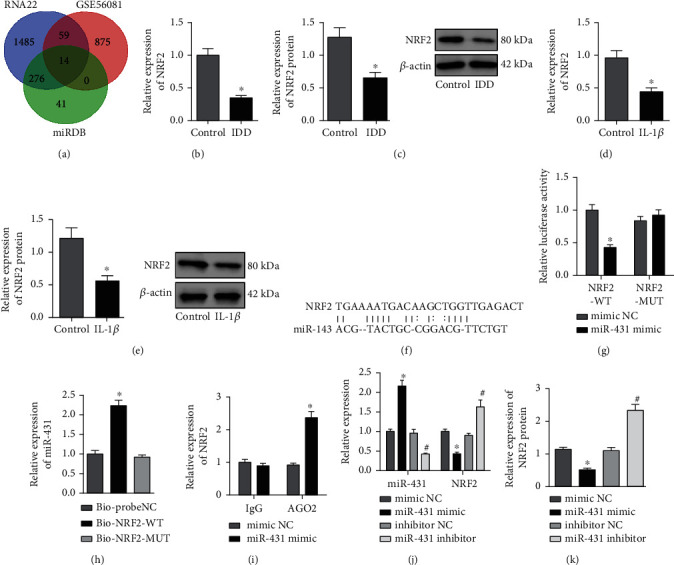
miR-431 targets and negatively regulates NRF2. (a) Prediction of the downstream target genes of miR-431. The green circle represents the downstream target gene predicted by miRDB database. The blue circle represents the downstream target gene predicted by RNA22 database. The red circle represents the significantly downregulated mRNA screened in the IDD sample of GSE56081 dataset. The middle part represents the intersection of three datasets. (b) NRF2 mRNA level in NP tissues of IDD mice determined by RT-qPCR (*n* = 10, ^∗^*p* < 0.05*vs.* control mice). (c) NRF2 protein level in NP tissues of IDD mice determined by Western blot analysis (*n* = 10, ^∗^*p* < 0.05*vs.* control mice). (d) NRF2 mRNA level in IL-1*β*-treated NPCs determined by RT-qPCR (^∗^*p* < 0.05*vs.* control NPCs). (e) NRF2 protein level in IL-1*β*-treated NPCs determined by Western blot analysis (^∗^*p* < 0.05*vs.* control NPCs). (f) The binding site between miR-431 and NRF2 predicted by miRDB database. (g) The relationship between miR-431 and NRF2 verified by dual-luciferase reporter gene assay (^∗^*p* < 0.05*vs.* mimic NC). (h) The relationship between miR-431 and NRF2 verified by RNA pull-down assay (^∗^*p* < 0.05*vs.* Bioprobe NC or Bio-NRF2-MUT). (i) The interaction between miR-431 and NRF2 detected by RIP assay (^∗^*p* < 0.05*vs.* mimic NC). (j) miR-431 expression and NRF2 mRNA level in NPCs in response to miR-431 mimic or miR-431 inhibitor determined by RT-qPCR (^∗^*p* < 0.05*vs.* NPCs transfected with mimic NC; ^#^*p* < 0.05*vs.* NPCs transfected with inhibitor NC). (k) NRF2 protein level in NPCs in response to miR-431 mimic or miR-431 inhibitor determined by Western blot analysis (^∗^*p* < 0.05*vs.* NPCs transfected with mimic NC; ^#^*p* < 0.05*vs.* NPCs transfected with inhibitor NC). Cell experiments were repeated for three times independently.

In order to further study the downstream mechanism, the target genes of miR-431 were predicted through RNA22 and miRDB databases. The predicted results of RNA22 and miRDB databases were intersected with the significantly downregulated mRNA in the IDD sample of GSE56081 (Figure 4(a)), revealing T13, DNMT3B, TSPYL4, DISC1, ZBTB4, PCNP, FAS, UBA3, PSME4, BTBD3, ADAM33, MICAL, and NRF2 in the intersection. A recent study has also demonstrated that NRF2 expression gradually decreased with the occurrence of IDD [26]. It was noted from PCR and Western blot analysis that NRF2 was poorly expressed in NP tissues of IDD mice (Figures 4(b) and 4(c)) and IL-1*β*-treated NPCs (Figures 4(d) and 4(e)).

Whether miR-431 regulated NRF2 was further explored. There was a binding site between miR-431 and NRF2 through miRDB database (Figure 4(f)), which was verified by dual-luciferase reporter gene assay. The luciferase activity of NRF2-3′UTR-WT was inhibited by overexpressed miR-431, while no evident difference was found with regard to NRF2-3′UTR-MUT (Figure 4(g)). RNA pull-down and RIP assay confirmed that there was interaction between miR-431 and NRF2 (Figures 4(h) and 4(i)). In addition, miR-431 expression was elevated, and NRF2 expression was reduced in NPCs transfected with miR-431 mimic, while the results were opposite in NPCs transfected with miR-431 inhibitor (Figures 4(j) and 4(k)).

These findings concluded that miR-431 targeted and suppressed NRF2 expression.

### 3.5. circ_0072464 Upregulated NRF2 by Sponging miR-431 to Inhibit NPC Ferroptosis

**Figure 5:**
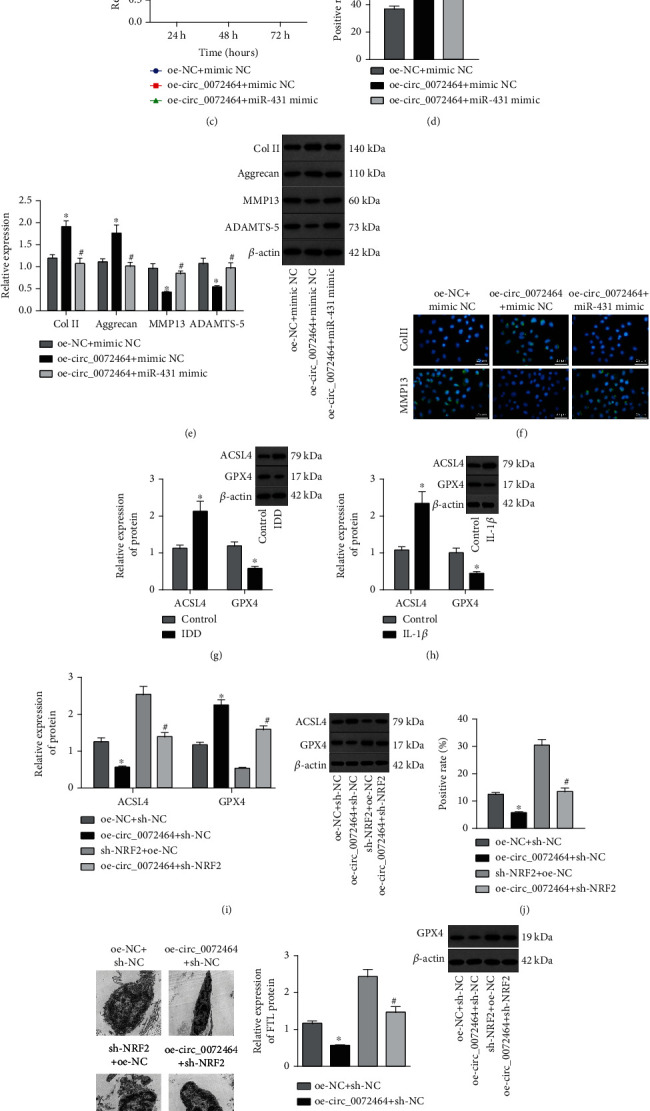
circ_0072464 affects NPC ferroptosis via the miR-431/NRF2 axis. (a) Expression of circ_0072464, miR-431, and NRF2 in NPCs transduced with oe-circ_0072464 alone or combined with miR-431 mimic determined by RT-qPCR (^∗^*p* < 0.05*vs.* NPCs transduced with oe-NC+mimic NC and ^#^*p* < 0.05*vs.* NPCs transduced with oe-circ_0072464+mimic NC). (b) NRF2 protein level in NPCs transduced with oe-circ_0072464 alone or combined with miR-431 mimic determined by Western blot analysis (^∗^*p* < 0.05*vs.* NPCs transduced with oe-NC+mimic NC and ^#^*p* < 0.05*vs.* NPCs transduced with oe-circ_0072464+mimic NC). (c) Viability of NPCs transduced with oe-circ_0072464 alone or combined with miR-431 mimic detected by CCK8 assay (^∗^*p* < 0.05*vs.* NPCs transduced with oe-NC+mimic NC and ^#^*p* < 0.05*vs.* NPCs transduced with oe-circ_0072464+mimic NC). (d) Proliferation of NPCs transduced with oe-circ_0072464 alone or combined with miR-431 mimic detected by EdU assay (^∗^*p* < 0.05*vs.* NPCs transduced with oe-NC+mimic NC and ^#^*p* < 0.05*vs.* NPCs transduced with oe-circ_0072464+mimic NC). (e) Protein levels of Col II, Aggrecan, MMP13, and ADAMTS-5 in NPCs transduced with oe-circ_0072464 alone or combined with miR-431 mimic measured by Western blot analysis (^∗^*p* < 0.05*vs.* NPCs transduced with oe-NC+mimic NC and ^#^*p* < 0.05*vs.* NPCs transduced with oe-circ_0072464+mimic NC). (f) Expression of Col II and MMP13 in NPCs transduced with oe-circ_0072464 alone or combined with miR-431 mimic determined by immunofluorescence staining. (g) Protein levels of GPX4 and ACSL4 in NP tissues of IDD mice determined by Western blot analysis (*n* = 10, ^∗^*p* < 0.05*vs.* control mice). (h) Protein levels of GPX4 and ACSL4 in IL-1*β*-treated NPCs determined by Western blot analysis (^∗^*p* < 0.05*vs.* control NPCs). (i) Protein levels of GPX4 and ACSL4 in NPCs transduced with oe-circ_0072464 and/or sh-NRF2 determined by Western blot analysis. (j) Cellular lipid ROS level of NPCs transduced with oe-circ_0072464 and/or sh-NRF2 detected by flow cytometry. (k) Mitochondrial morphology of NPCs transduced with oe-circ_0072464 and/or sh-NRF2 observed under the TEM. (l) FTL protein level in NPCs transduced with oe-circ_0072464 and/or sh-NRF2 measured by Western blot analysis. (m) Measurement of free iron levels in NPCs transduced with oe-circ_0072464 and/or sh-NRF2. In (e–i), ^∗^*p* < 0.05*vs.* NPCs transduced with oe-NC+sh-NC; ^#^*p* < 0.05*vs.* NPCs transduced with oe-circ_0072464+sh-NC. Cell experiments were repeated for three times independently.

It has been demonstrated that ferroptosis can promote the death of AF cells and NPCs to mediate oxidative stress response, thereby promoting IDD [27]. Therefore, we further studied whether circ_0072464 regulated ferroptosis by upregulating the miR-431/NRF2 axis. The expression of circ_0072464 and NRF2 was increased, and miR-431 expression was deceased in NPCs transduced with oe-circ_0072464, while further treatment of miR-431 mimic resulted in no obvious difference in circ_0072464 expression but elevated miR-431 and diminished NRF2 (Figures 5(a) and 5(b)).

As shown in Figures 5(c) and 5(d), cell proliferation was augmented upon circ_0072464 overexpression while it was reduced in the presence of further miR-431 overexpression. In addition, the results of Western blot analysis and immunofluorescence staining suggested an increase in the expression of Col II and Aggrecan yet a reduction in that of MMP13 and ADAMTS-5 in NPCs transduced with oe-circ_0072464 while opposite results were found in response to oe-circ_0072464+miR-431 mimic treatment (Figures 5(e) and 5(f)).

The effects of circ_0072464 regulating miR-431/NRF2 on NPC ferroptosis were explored. ACSL4 expression was elevated, and GPX4 expression was decreased in NP tissues of IDD mice (Figure 5(g)) and IL-1*β*-treated NPCs (Figure 5(h)). Next, NPCs were overexpressing circ_0072464 and/or sh-NRF2. It was observed that ACSL4 expression was reduced, and GPX4 expression was increased in NPCs overexpressing circ_0072464, while the results were opposite in NPCs transduced with sh-NRF2. In NPCs overexpressing circ_0072464, sh-NRF2 led to elevated ACSL4 expression and diminished GPX4 expression (Figure 5(i)).

In addition, flow cytometry, TEM, Western blot analysis, and labile iron detection results (Figures 5(j)–5(m)) presented that the content of lipid ROS was decreased, the morphology of mitochondria was intact, and the levels of FTL and intracellular free iron were decreased in NPCs overexpressing circ_0072464. After silencing of NRF2, the content of lipid ROS in NPCs was increased, the mitochondria were damaged, and the levels of FTL and intracellular free iron were increased. Moreover, the content of lipid ROS in NPCs was increased, the mitochondria were seriously damaged, and the levels of FTL and intracellular free iron were obviously increased in NPCs in response to overexpression of circ_0072464 and silencing of NRF2.

These findings demonstrated that circ_0072464 promoted NPC proliferation and matrix synthesis while inhibiting NPC ferroptosis by sponging miR-431 and upregulating NRF2.

### 3.6. circ_0072464 Inhibited NPC Ferroptosis to Promote Matrix Synthesis and Proliferation of NPCs via miR-431/NRF2

**Figure 6:**
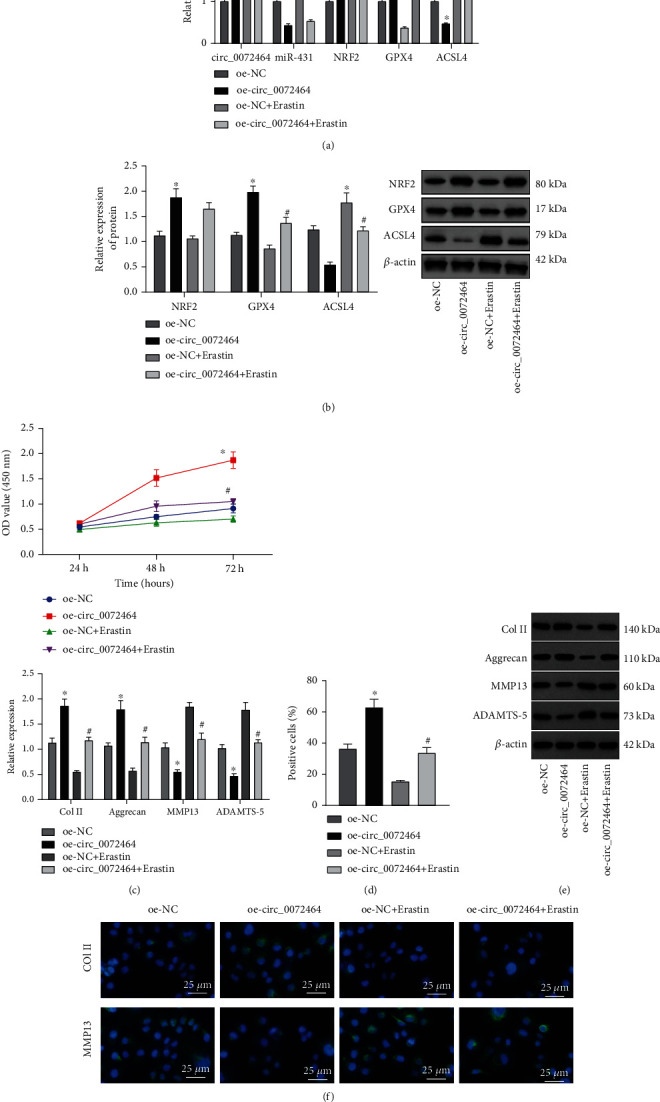
circ_0072464/miR-431/NRF2 affects matrix synthesis and proliferation of NPCs by regulating NPC ferroptosis. (a) Expression of circ_0072464, miR-431, NRF2, GPX4, and ACSL4 in NPCs in response to oe-circ_0072464 and/or Erastin determined by RT-qPCR. (b) Protein levels of NRF2, GPX4, and ACSL4 in NPCs in response to oe-circ_0072464 and/or Erastin determined by Western blot analysis. (c) NPC proliferation detected by CCK8 assay. (d) NPC proliferation in response to oe-circ_0072464 and/or Erastin detected by EdU assay. (e) Protein levels of Col II, Aggrecan, MMP13, and ADAMTS-5 in NPCs in response to oe-circ_0072464 and/or Erastin determined by Western blot analysis. (f) Expression of Col II, Aggrecan, MMP13, and ADAMTS-5 in NPCs in response to oe-circ_0072464 and/or Erastin determined by immunofluorescence staining. ^∗^*p* < 0.05*vs.* NPCs transduced with oe-NC; ^#^*p* < 0.05*vs.* NPCs transduced with oe-circ_0072464. Cell experiments were repeated for three times independently.

Furthermore, NPCs were treated with oe-circ_0072464 and/or Erastin to examine the effects of circ_0072464/miR-431/NRF2 axis on cell activities of NPCs. RT-qPCR and Western blot analysis showed that expression of circ_0072464, NRF2, and GPX4 was elevated, and expression of miR-431 and ACSL4 was reduced after overexpression of circ_0072464. Additional treatment of Erastin led to diminished GPX4 expression and elevated ACSL4 expression, but expression of circ_0072464, miR-431, and NRF2 displayed no evident difference. Relative to oe-circ_0072464 alone, GPX4 expression was decreased, and ACSL4 expression was increased upon cotreatment of oe-circ_0072464 and Erastin (Figures 6(a) and 6(b)).

Subsequently, CCK8 assay and EdU assay exhibited that overexpression of circ_0072464 enhanced NPC proliferation, and Erastin suppressed NPC proliferation. Besides, proliferation was repressed by Erastin in NPCs overexpressing circ_0072464 (Figures 6(c) and 6(d)). Western blot analysis and immunofluorescence staining showed that circ_0072464 overexpression elevated levels of Col II and Aggrecan and reduced levels of MMP13 and ADAMTS-5 in NPCs, while Erastin exerted the opposite effects. Simultaneous treatment with oe-circ_0072464 and Erastin decreased levels of Col II and Aggrecan and increased levels of MMP13 and ADAMTS-5 in NPCs in comparison to oe-circ_0072464 alone (Figures 6(e) and 6(f)). Thus, Erastin-induced ferroptosis inhibited the facilitating role of circ_0072464 in matrix synthesis and proliferation of NPCs.

The obtained data confirmed that circ_0072464 repressed NPC ferroptosis to enhance matrix synthesis and proliferation of NPCs by regulating the miR-431/NRF2 axis.

### 3.7. circ_0072464 Delivered by BMSC-EVs Alleviated IVD Lesions in IDD Mice through miR-431/NRF2

At last, whether circ_0072464-containing BMSC-EVs inhibit NPC ferroptosis to relieve IDD via the miR-431/NRF2 was validated *in vivo*. IDD mice were injected with EVs and/or Erastin, and NP tissues were isolated for further experiments.

**Figure 7:**
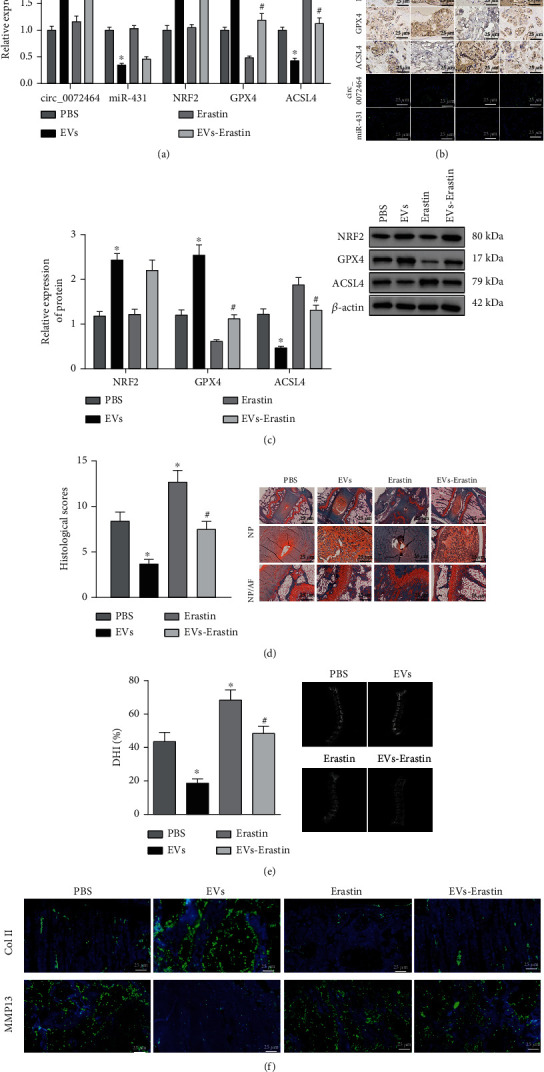
BMSC-EV-packaged circ_0072464 regulates miR-431/NRF2 to affect NPC ferroptosis in IDD mice. IDD mice were injected with EVs and/or Erastin (*n* = 10). (a) Expression of circ_0072464, miR-431, NRF2, GPX4, and ACSL4 in NP tissues of IDD mice measured by RT-qPCR. (b) Expression of NRF2, GPX4, and ACSL4 in NP tissues of IDD mice detected by immunohistochemistry, and expression of circ_0072464 (green fluorescence) and miR-431 (green fluorescence) in NP tissues of IDD mice detected by RNA FISH. (c) Protein levels of NRF2, GPX4, and ACSL4 in NP tissues of IDD mice determined by Western blot analysis. (d) Pathological changes of IVD, NP, and AF in IDD mice detected by safranin O staining. The first row shows the staining image of IVD as a whole, the second row displays the detailed view of NP tissues, and the third row presents the detailed view of boundary between NP and AF tissues. (e) Degeneration of IVD in IDD mice observed using X-ray. (f) Levels of Col II and MMP13 in NP tissues of IDD mice detected by immunofluorescence staining. ^∗^*p* < 0.05*vs.* IDD mice injected with PBS; ^#^*p* < 0.05*vs.* IDD mice injected with EVs.

We found elevations in expression of circ_0072464, NRF2, and GPX4 and reductions in expression of miR-431 and ACSL4 in NP tissues of IDD mice injected with EVs, while GPX4 expression reduced and ACSL4 expression elevated in NP tissues of IDD mice injected with Erastin. Compared with IDD mice injected with EVs, GPX4 expression decreased and ACSL4 expression increased in NP tissues of IDD mice injected with EVs+Erastin (Figures 7(a)–7(c)).

Safranin O staining and X-ray exhibited that the degree of morphological disorder of NP and AF and the degeneration of IVD were reduced in IDD mice injected with EVs, while the results were opposite in IDD mice injected with Erastin. Compared with IDD mice injected with EVs, the degree of morphological disorder and degeneration of NP and AF were also aggravated in IDD mice injected with EVs+Erastin (Figures 7(d) and 7(e)). In addition, immunofluorescence staining presented that MMP13 expression decreased and Col II expression increased in NP tissues of IDD mice injected with EVs, while Erastin exerted the opposite effects. Compared with IDD mice injected with EVs, MMP13 expression elevated and Col II expression decreased in NP tissues of IDD mice injected with EVs+Erastin (Figure 7(f)).

These findings demonstrated that the transfer of circ_0072464 by BMSC-EVs regulated the miR-431/NRF2 axis to repress NPC ferroptosis, thereby alleviating IVD lesions in IDD mice.

## 4. Discussion

In recent years, MSC-secreted EVs have emerged as a potential therapeutic strategy for IDD [28]. The data obtained in our study demonstrated that circ_0072464 shuttled by BMSC-derived EVs was transferred into NPCs, where circ_0072464 suppressed NPC ferroptosis to relieve IDD by regulating miR-431/NRF2 both *in vitro* and *in vivo*.

Evidence accumulates on the biological intervention of BMSCs in IDD, suggesting a potential for identifying suitable biotherapeutic targets [29]. EVs have been documented to be secreted by BMSCs, which provide therapeutic benefits for IDD [30]. Our findings confirmed that the transfer of BMSCs-EVs to NPCs alleviated IDD in mice, as reflected by decreased MMP13 level and increased Col II level. MMP13 and Col II are known to reflect the synthesis of IVD matrix during IDD [24, 25, 31]. Consistently, a recent study has also demonstrated that BMSC-derived EVs protect IVD against degeneration by reducing levels of expression of Col II and Aggrecan while inhibiting NPC apoptosis [15].

Moreover, we reported that circ_0072464 shuttled by BMSC-EVs promoted matrix synthesis and proliferation of NPCs through inhibiting NPC ferroptosis. NPCs are capable of inducing the generation of extracellular matrix for maintaining the hydration state of IVD [32]. The typical feature of IDD includes the degradation of NP and AF matrix components, which impairs the bearing performance of IVD [33]. Therefore, the alleviatory effect of circ_0072464 could be confirmed. In addition, our data also demonstrated that circ_0072464 competitively binds to miR-431, with downregulated circ_0072464 and upregulated miR-431 observed in IDD. circRNAs serve as miRNA sponges and ceRNAs to interact with miRNAs at a posttranscriptional level, thus downregulating miRNA and upregulating miRNA downstream targets [34]. The binding between circRNAs and miRNAs regulates cell proliferation, mitophagy, and apoptosis in IDD [10]. Of note, miR-431-5p has been documented to be upregulated in IDD and circARL15 acts as a ceRNA to reduce miR-431-5p expression, thus inhibiting NPC apoptosis but inducing NPC proliferation to alleviate IDD [35], providing validation of our finding.

Further mechanistic explorations in this study confirmed that miR-431 targeted and repressed NRF2, which was poorly expressed in IDD. Evidence exists reporting that NRF2 expression is decreased in NP tissues of patients IDD [26]. The important roles of NRF2 in alleviating lipid peroxidation and suppressing ferroptosis have also been verified [27]. Available evidence has indicated that ferroptosis of NPCs is implicated in pathogenesis of IDD, suggesting that targeting NPC ferroptosis may be a novel target for treating IDD [36]. Both *in vitro* and *in vivo* experiments in the present study confirmed that circ_0072464 shuttled by BMSC-secreted EVs inhibits NPC ferroptosis by inducing matrix synthesis and proliferation of NPCs to relieve IDD via downregulation of miR-431 and upregulation of NRF2.

**Figure 8:**
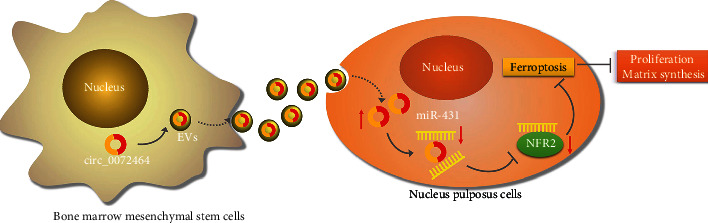
Molecular mechanism of the effects of circ_0072464 shuttled by BMSCs-EVs on IDD by regulating NPC ferroptosis via miR-431/NRF2. circ_0072464 shuttled by BMSCs-EVs upregulates NRF2 expression by competitively binding to miR-431, thereby inhibiting ferroptosis and promoting matrix synthesis and proliferation of NPCs, ultimately relieving IDD.

To sum up, our study demonstrated that circ_0072464 transferred by BMSC-derived EVs upregulated NRF2 expression through competitive binding to miR-431, the mechanism of which led to inhibition of NPC ferroptosis and alleviation of IDD (Figure 8). Our findings pave way for the development of potential therapeutic strategies for inhibiting NPC ferroptosis to alleviate IDD. Due to the limited known researches on the functional role of circ_0072464, the underlying mechanism concerning circ_0072464 shuttled by BMSC-derived EVs, miR-431, and NRF2 should be more clearly investigated in the future. More importantly, some other differentially expressed cicrRNAs in IDD were revealed from our RNA-seq results in addition to circ_0072464, highly suggestive of the need to identify the potential therapeutic implication of novel cicrRNAs in IDD. Meanwhile, the administration and dosage of agents to inhibit ferroptosis process are urgently required to be determined to improve treatment efficacy against IDD.

## Data Availability

The datasets generated for this study are available on request to the corresponding author.

## Supplementary Materials

Supplementary Materials Figure S1: isolation of NP tissues and identification of NPCs. Figure S2: identification of mouse BMSCs. Figure S3: circ_0072464 and miR-431 have strong homology in humans and mice. Table S1: primer sequences of RT-qPCR.

## References

[1] Yang S., Zhang F., Ma J., Ding W. (2020). Intervertebral disc ageing and degeneration: the antiapoptotic effect of oestrogen. *Ageing Research Reviews*.

[2] Priyadarshani P., Li Y., Yao L. (2016). Advances in biological therapy for nucleus pulposus regeneration. *Osteoarthritis and Cartilage*.

[3] Desmoulin G. T., Pradhan V., Milner T. E. (2020). Mechanical aspects of intervertebral disc injury and implications on biomechanics. *Spine*.

[4] Lu S., Song Y., Luo R. (2021). Ferroportin-dependent iron homeostasis protects against oxidative stress-induced nucleus pulposus cell ferroptosis and ameliorates intervertebral disc degeneration in vivo. *Oxidative Medicine and Cellular Longevity*.

[5] Zhang X., Huang Z., Xie Z. (2020). Homocysteine induces oxidative stress and ferroptosis of nucleus pulposus via enhancing methylation of GPX4. *Free Radical Biology & Medicine*.

[6] Xia C., Zeng Z., Fang B. (2019). Mesenchymal stem cell-derived exosomes ameliorate intervertebral disc degeneration via anti-oxidant and anti-inflammatory effects. *Free Radical Biology & Medicine*.

[7] Li Y., Zhao J., Yu S. (2019). Extracellular vesicles long RNA sequencing reveals abundant mRNA, circRNA, and lncRNA in human blood as potential biomarkers for cancer diagnosis. *Clinical Chemistry*.

[8] DiStefano T. J., Vaso K., Danias G., Chionuma H. N., Weiser J. R., Iatridis J. C. (2022). Extracellular vesicles as an emerging treatment option for intervertebral disc degeneration: therapeutic potential, translational pathways, and regulatory considerations. *Advanced Healthcare Materials*.

[9] Song J., Chen Z. H., Zheng C. J. (2020). Exosome-transported circRNA_0000253 competitively adsorbs microRNA-141-5p and increases IDD. *Molecular Therapy-Nucleic Acids*.

[10] Li Z., Chen X., Xu D., Li S., Chan M. T. V., Wu W. K. K. (2019). Circular RNAs in nucleus pulposus cell function and intervertebral disc degeneration. *Cell Proliferation*.

[11] Huo Z., Li H., Tian L. (2021). Construction of a potentially functional circRNA-miRNA-mRNA network in intervertebral disc degeneration by bioinformatics analysis. *BioMed Research International*.

[12] Cazzanelli P., Wuertz-Kozak K. (2020). MicroRNAs in intervertebral disc degeneration, apoptosis, inflammation, and mechanobiology. *International Journal of Molecular Sciences*.

[13] Zhou X., Chen L., Grad S. (2017). The roles and perspectives of microRNAs as biomarkers for intervertebral disc degeneration. *Journal of Tissue Engineering and Regenerative Medicine*.

[14] Wang X., Wang B., Zou M. (2018). CircSEMA4B targets miR-431 modulating IL-1beta-induced degradative changes in nucleus pulposus cells in intervertebral disc degeneration via Wnt pathway. *Biochimica et Biophysica Acta - Molecular Basis of Disease*.

[15] Wen T., Wang H., Li Y. (2021). Bone mesenchymal stem cell-derived extracellular vesicles promote the repair of intervertebral disc degeneration by transferring microRNA-199a. *Cell Cycle*.

[16] Soleimani M., Nadri S. (2009). A protocol for isolation and culture of mesenchymal stem cells from mouse bone marrow. *Nature Protocols*.

[17] Maridas D. E., Rendina-Ruedy E., Le P. T., Rosen C. J. (2018). Isolation, culture, and differentiation of bone marrow stromal cells and osteoclast progenitors from mice. *Journal of Visualized Experiments*.

[18] Ji M. L., Jiang H., Zhang X. J. (2018). Preclinical development of a microRNA-based therapy for intervertebral disc degeneration. *Nature Communications*.

[19] Li X. N., Wang Z. J., Ye C. X., Zhao B. C., Li Z. L., Yang Y. (2018). RNA sequencing reveals the expression profiles of circRNA and indicates that circDDX17 acts as a tumor suppressor in colorectal cancer. *Journal of Experimental & Clinical Cancer Research*.

[20] Gao Y., Wang J., Zhao F. (2015). CIRI: an efficient and unbiased algorithm for de novo circular RNA identification. *Genome Biology*.

[21] Ritchie M. E., Phipson B., Wu D. (2015). limma powers differential expression analyses for RNA-sequencing and microarray studies. *Nucleic Acids Research*.

[22] Sun J., Tian X., Zhang J. (2017). Regulation of human glioma cell apoptosis and invasion by miR-152-3p through targeting DNMT1 and regulating NF2: miR-152-3p regulate glioma cell apoptosis and invasion. *Journal of Experimental & Clinical Cancer Research*.

[23] Cheng X., Zhang L., Zhang K. (2018). Circular RNA VMA21 protects against intervertebral disc degeneration through targeting miR-200c and X linked inhibitor-of-apoptosis protein. *Annals of the Rheumatic Diseases*.

[24] Wang X., Peng L., Gong X., Zhang X., Sun R., Du J. (2018). LncRNA-RMRP promotes nucleus pulposus cell proliferation through regulating miR-206 expression. *Journal of Cellular and Molecular Medicine*.

[25] Choi H., Tessier S., Silagi E. S. (2018). A novel mouse model of intervertebral disc degeneration shows altered cell fate and matrix homeostasis. *Matrix Biology*.

[26] Tang Z., Hu B., Zang F., Wang J., Zhang X., Chen H. (2019). Nrf2 drives oxidative stress-induced autophagy in nucleus pulposus cells via a Keap1/Nrf2/p62 feedback loop to protect intervertebral disc from degeneration. *Cell Death & Disease*.

[27] Dodson M., Castro-Portuguez R., Zhang D. D. (2019). NRF2 plays a critical role in mitigating lipid peroxidation and ferroptosis. *Redox Biology*.

[28] Sun Y., Zhang W., Li X. (2021). Induced pluripotent stem cell-derived mesenchymal stem cells deliver exogenous miR-105-5p via small extracellular vesicles to rejuvenate senescent nucleus pulposus cells and attenuate intervertebral disc degeneration. *Stem Cell Research & Therapy*.

[29] Hingert D., Ekstrom K., Aldridge J., Crescitelli R., Brisby H. (2020). Extracellular vesicles from human mesenchymal stem cells expedite chondrogenesis in 3D human degenerative disc cell cultures. *Stem Cell Research & Therapy*.

[30] Hu Z. L., Li H. Y., Chang X. (2020). Exosomes derived from stem cells as an emerging therapeutic strategy for intervertebral disc degeneration. *World Journal of Stem Cells*.

[31] Liu W., Xia P., Feng J. (2017). MicroRNA-132 upregulation promotes matrix degradation in intervertebral disc degeneration. *Experimental Cell Research*.

[32] Zhang J., Zhang J., Zhang Y. (2020). Mesenchymal stem cells-derived exosomes ameliorate intervertebral disc degeneration through inhibiting pyroptosis. *Journal of Cellular and Molecular Medicine*.

[33] Ashinsky B. G., Bonnevie E. D., Mandalapu S. A. (2020). Intervertebral disc degeneration is associated with aberrant endplate remodeling and reduced small molecule transport. *Journal of Bone and Mineral Research*.

[34] Du W. W., Zhang C., Yang W., Yong T., Awan F. M., Yang B. B. (2017). Identifying and characterizing circRNA-protein interaction. *Theranostics*.

[35] Wang H., Zhu Y., Cao L., Sun K., Qiu W., Fan H. (2021). circARL15 plays a critical role in intervertebral disc degeneration by modulating miR-431-5p/DISC1. *Frontiers in Genetics*.

