# Supplementary material for: circ_0072464 Shuttled by Bone Mesenchymal Stem Cell-Secreted Extracellular Vesicles Inhibits Nucleus Pulposus Cell Ferroptosis to Relieve Intervertebral Disc Degeneration
Source: Oxid Med Cell Longev. 2022 Jun 29;2022:2948090. doi: 10.1155/2022/2948090 (PMC9259290; doi:10.1155/2022/2948090)
Supplement: Supplementary Materials — Figure S1: isolation of NP tissues and identification of NPCs. Figure S2: identification of mouse BMSCs. Figure S3: circ_0072464 and miR-431 have strong homology in humans and mice. Table S1: primer sequences of RT-qPCR. [file 2948090.f1.docx]

**
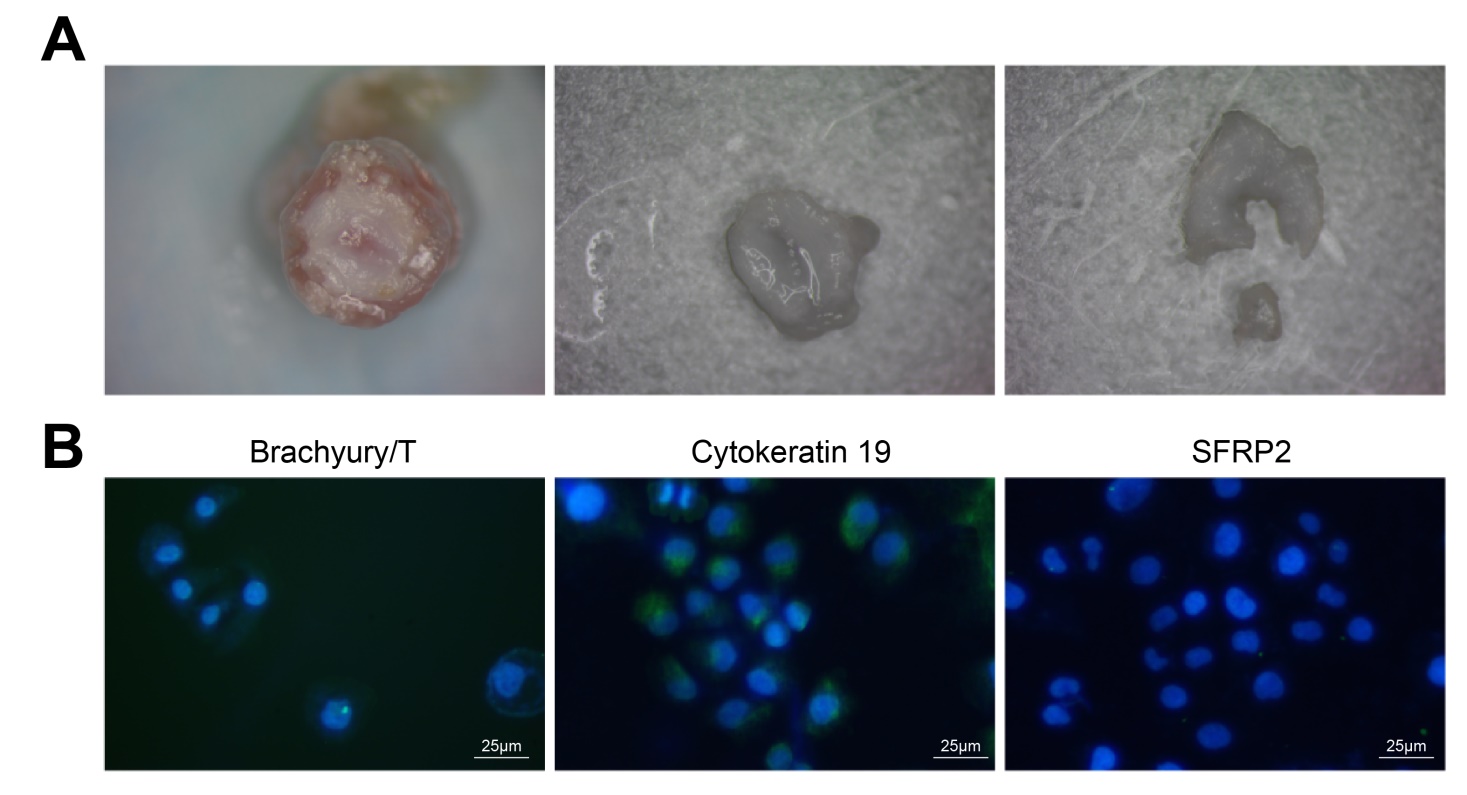
**

**FIGURE S1. Isolation of NP tissues and identification of NPCs. A,** Localization of NP tissues shown by IVD cross section. **B,** Expression of Brachyury/T and Cytokeratin 19 (positive for NPCs) and SFRP2 (positive for AF cells, mainly localized in the cytoplasm of mice) detected by immunofluorescence assay.

**
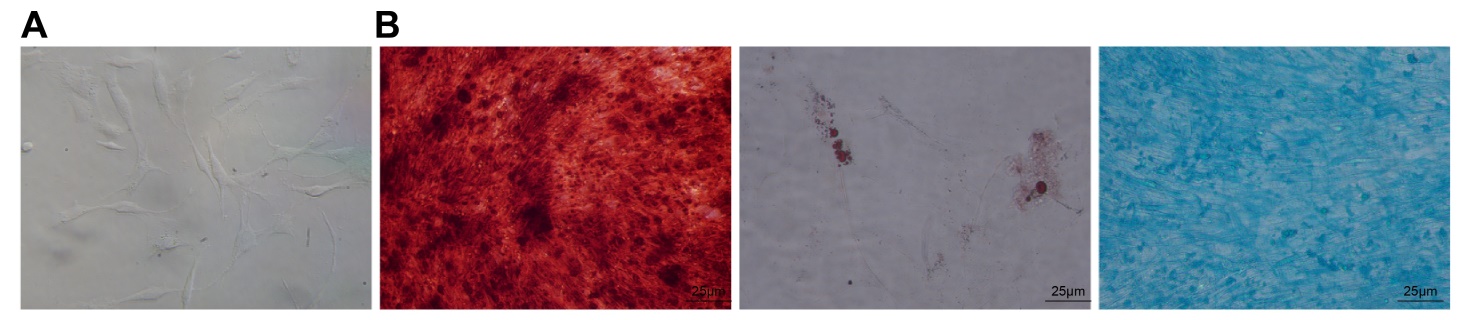
**

**FIGURE S2.** **Identification of mouse BMSCs.** **A**, The morphology of isolated BMSCs on the 5^th^ day of incubation observed under the inverted microscope. **B**, The differentiation of BMSCs into osteoblasts, adipoblasts and chondroblasts analyzed by alizarin red staining (left panel), oil red O staining (middle panel) and alcian blue staining (right panel), respectively.

**
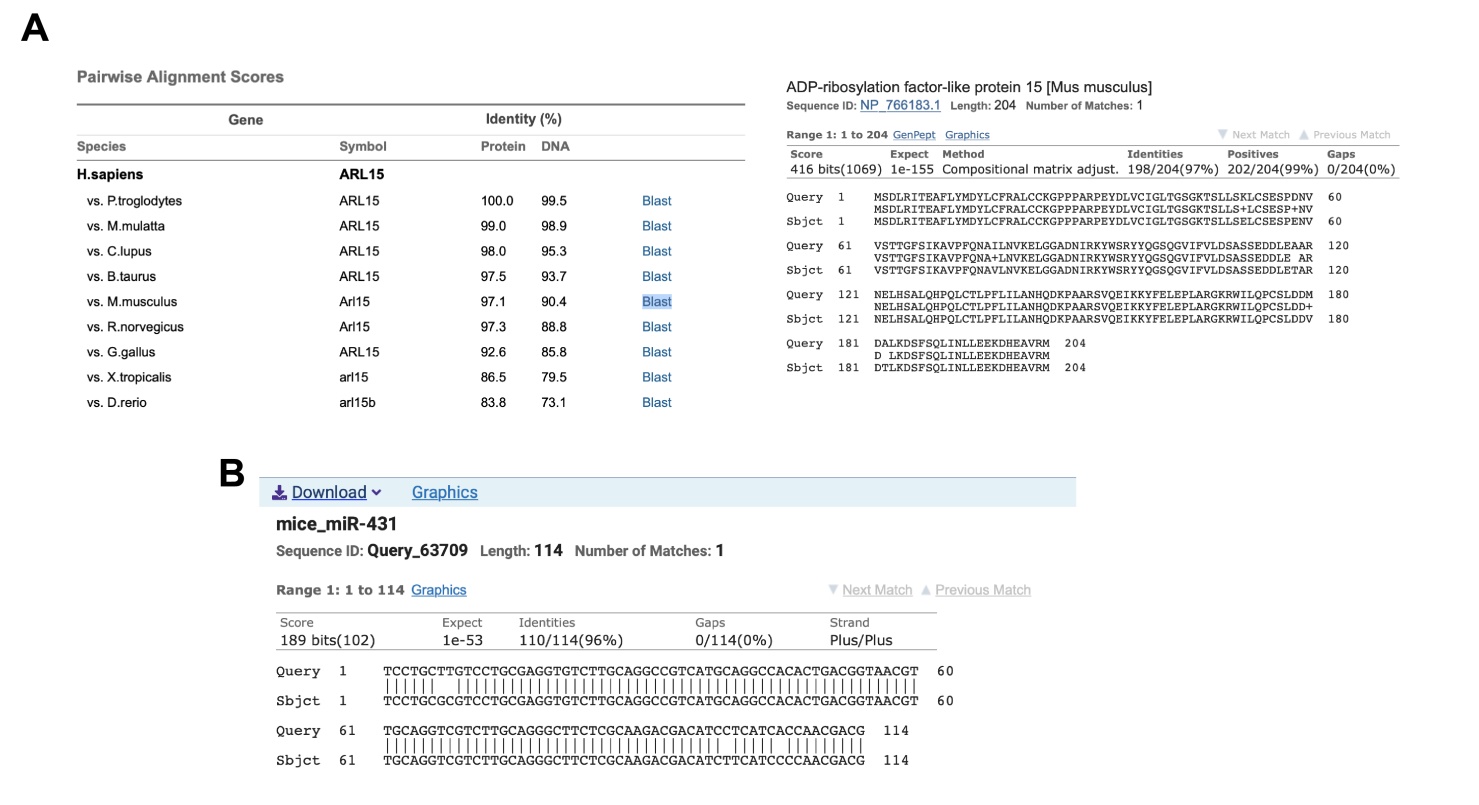
**

**FIGURE S3.** **circ_0072464 and miR-431 have strong homology in humans and mice. A,** BLAST comparative analysis showing circ_0072464 (circARL15) encoded by ARL15 both in human and mice. **B,** BLAST comparative analysis on human and murine miR-431 sequences.

**Table S1.** Primer sequences of RT-qPCR

| Genes | Forward (5’ - 3’) | Reverse (5’ - 3’) |
| --- | --- | --- |
| circ_0072464 (mouse) | CTCATAAACTTGTTAGAAGAA | GCTGGCTTGTCTTGATGATTGG |
| miR-431 (mouse) | GCGTGTCTTGCAGGCCGT | Universal reverse primer |
| NRF2 (mouse) | TGAAGCTCAGCTCGCATTGA | TGCTCCAGCTCGACAATGTT |
| ACSL4 (mouse) | CCACACTTATGGCCGCTGTT | GGGCGTCATAGCCTTTCTTG |
| GPX4 (mouse) | GCAGGAGCCAGGAAGTAATC | GGCTGGACTTTCATCCATTT |
| U6 (mouse) | GCTTCGGCAGCACATATACT | Universal reverse primer |
| β-actin (mouse) | AGAGGGAAATCGTGCGTGAC | CAATAGTGATGACCTGGCCGT |

Note: RT-qPCR, reverse transcription quantitative polymerase chain reaction; miR, microRNA; NRF2, nuclear factor E2-related factor 2; ACSL4, acyl-CoA synthetase long-chain family member 4; GPX4, glutathione peroxidase 4.
